# On the Comparative Advantages of Wide and Narrow Plates for Artificial Teeth

**Published:** 1851-07

**Authors:** J. W. Crane

**Affiliations:** New York.


### ARTICLE VIII.

### *On the Comparative Advantages of Wide and Narrow Plates for Artificial Teeth.* By J. W. CRANE, M. D., *New York.*

MESSRS. EDITORS :—I proposed in my last communication, to give some further hints on the subject of using wide plates for the purpose of adhesion, but sickness, and death in my family, has prevented me from doing it in due time.

I have never heard any argument in favor of covering the whole surface of the palatine portion of the mouth, except *to produce greater adhesion.* I have never had occasion to use a wide plate for that or any other purpose, except, where the palatine plate of bone was wanting, and then, it was used for the purpose of correcting the articulation in speaking.

Not long after these plates came into use, I had occasion to reduce their width to make them comfortable, or to throw them aside altogether, and make others of less width. This was sufficient to convince me, that the new plan was inferior to the one I had adopted.

I know of no better plan to test the utility of any, so called improvement, than to weigh the arguments in favor, and against it. We have already mentioned the only argument, that we know of, in favor of the wide plate, viz. *to produce greater adhesion.*

In the first place, does it produce the desired object, more effectually than the narrow plate with a chamber, under the linings of the teeth ?

2d. Which is the most permanent in its action ?

3d. If the narrow plate injures the taste, will not the wide one destroy it ?

1st. I have never seen a wide plate adhere to the gum so firmly as the narrow one, with the chamber under the linings, which I have used since 1836. Some (appear to) use the wide plate for the purpose of distorting the anatomical form of the mouth by a chamber nearly in the centre of the plate ; others bend the posterior edge of the wide plate, and form a partial chamber of nearly its whole surface.

2d. The wide plate must, of necessity, be temporary, in what it is designed to accomplish. No one has ever seen a mouth, after the teeth were extracted, that did not change by absorption, and they will continue to do so for years ; after the first set are inserted, especially in the young subject, they change more or less after the teeth have been removed ten years. The absorption takes place on the alveoli, and not on the osa palati where the wide plate rests. This bone never absorbs except when diseased, and must therefore be the only resting place of the wide plate, when the alveoli has retired.

3d. Taste is injured, in some cases, by a plate one-fourth of an inch in width, when it has been carried back to the first molar tooth. There are persons who have lost their taste without any artificial means, by some physical derangement of the organs of taste. There are others who care but little on that subject, so long as the food keeps the soul and body united ; but there are not a few whose taste has been destroyed (if we can rely upon their own testimony) by the wide plate.

Physiology teaches us that the sense of taste is seated chiefly in the nervous papillæ of the tongue, which are excited into action by contact with the palatine portion of the mouth.

The sense of taste is undoubtedly combined with the sense of touch, and if the latter is destroyed by the interposition of a foreign substance, how much more is the former affected by the same cause.

